# New Insights into *Pseudomonas* spp.-Produced Antibiotics: Genetic Regulation of Biosynthesis and Implementation in Biotechnology

**DOI:** 10.3390/antibiotics13070597

**Published:** 2024-06-27

**Authors:** Alexandra Baukova, Alexander Bogun, Svetlana Sushkova, Tatiana Minkina, Saglara Mandzhieva, Ilya Alliluev, Hanuman Singh Jatav, Valery Kalinitchenko, Vishnu D. Rajput, Yanina Delegan

**Affiliations:** 1 Institute of Biochemistry and Physiology of Microorganisms, Federal Research Center “Pushchino Scientific Center for Biological Research of Russian Academy of Sciences” (FRC PSCBR RAS), 142290 Pushchino, Moscow Region, Russia; baukova_26@mail.ru (A.B.); bogun62@mail.ru (A.B.); 2 Pushchino Branch of Federal State Budgetary Educational Institution of Higher Education “Russian Biotechnology University (ROSBIOTECH)”, 142290 Pushchino, Moscow Region, Russia; 3 Academy of Biology and Biotechnology behalf D.I. Ivanovskyi, Southern Federal University, 344006 Rostov-on-Don, Russia; terra_rossa@mail.ru (S.S.); tminkina@mail.ru (T.M.); msaglara@mail.ru (S.M.); alliluev@sfedu.ru (I.A.); rajput.vishnu@gmail.com (V.D.R.); 4 Soil Science & Agricultural Chemistry, S.K.N. Agriculture University-Jobner, Jaipur 303329, Rajasthan, India; hsjatav.soils@sknau.ac.in; 5 Institute of Fertility of Soils of South Russia, 346493 Persianovka, Rostov Region, Russia; kalinitch@mail.ru; 6 All-Russian Research Institute for Phytopathology of the Russian Academy of Sciences, Institute St., 5, 143050 Big Vyazyomy, Moscow Region, Russia

**Keywords:** *Pseudomonas*, antibiotic production, mupirocin, pyrrolnitrin, DAPG, gluconic acid, genetic organization, biosynthesis pathways

## Abstract

*Pseudomonas* bacteria are renowned for their remarkable capacity to synthesize antibiotics, namely mupirocin, gluconic acid, pyrrolnitrin, and 2,4-diacetylphloroglucinol (DAPG). While these substances are extensively employed in agricultural biotechnology to safeguard plants against harmful bacteria and fungi, their potential for human medicine and healthcare remains highly promising for common science. However, the challenge of obtaining stable producers that yield higher quantities of these antibiotics continues to be a pertinent concern in modern biotechnology. Although the interest in antibiotics of *Pseudomonas* bacteria has persisted over the past century, many uncertainties still surround the regulation of the biosynthetic pathways of these compounds. Thus, the present review comprehensively studies the genetic organization and regulation of the biosynthesis of these antibiotics and provides a comprehensive summary of the genetic organization of antibiotic biosynthesis pathways in pseudomonas strains, appealing to both molecular biologists and biotechnologists. In addition, attention is also paid to the application of antibiotics in plant protection.

## 1. Introduction

Numerous soil bacteria have the ability to generate secondary metabolites that exhibit bacteriostatic and bactericidal properties. This enables them to thrive and maintain their competitiveness in demanding microbiome habitats [1,2,3]. *Pseudomonas* bacteria are renowned for their ability to produce a wide variety of antibiotic secondary metabolites, solidifying their reputation as some of the most prolific contributors to the field of microbial drug discovery [4,5]. The variety of compounds produced by *Pseudomonas* strains is mind-boggling.

*Pseudomonas* strains’ antibiotics play a crucial role in plant protection biotechnology by serving as antibacterial and antifungal agents [6,7]. Among them, fluorescent pseudomonads that produce phenazine co-compounds are the most commonly utilized [8]. Commercial products based on *P. aureofaciens*, *P. chlororaphis*, *P. fluorescens*, and *P. syringae* are widely recognized [9,10,11]. Various research papers offer an overview of the fluorescent *Pseudomonas* strain-based formulations used for plant protection in Europe, Australia, and the USA [12,13,14,15]. Typically, these strains were effective against pathogenic fungi, with some cases showing success against other pseudomonads [16]. One notable example was *Pseudomonas synxantha* PS54 [17], which was patented as a control agent against *P. tolaasii* [18], the causative agent of bacterial blotch in the mushroom *Agaricus bisporus* [19,20].

In the 20th century, antibiotics produced by *Pseudomonas* strains were utilized for human therapy, in addition to plant protection biotechnology. The strain *Pseudomonas lindbergii* (ATCC-31099) [21] was patented as a producer of antifungal compounds, which were claimed to be more effective than griseofulvin, an antibiotic widely used by the USA Army against fungal infections. To demonstrate the efficacy of the *P. lindbergii* antibiotics, they were put on filter paper and placed on the feet and infected toenails of patients. It was claimed that the manifestations of infection disappeared after 2–3 days.

Additionally, *P. lindbergii* and *Bacillus coagulans* have been combined in preparations for controlling mycoses, showing promising results [22,23]. For prophylaxis or therapeutic treatment, the clean and dry feet of patients were treated with a preparation containing supernatant or filtrate of *B. coagulans* or *P. lindbergii*. In addition to fungi, antibiotics produced by *P. lindbergii* were effective against *Streptococcus*, *Staphylococcus*, *E. coli*, *Serratia*, *Klebsiella* and *Proteus* [21].

Phenazine antibiotics, a group of compounds produced by *Pseudomonas* strains, have been extensively studied. The production of these antibiotics involved genetic and technological aspects, highlighting the importance of a complete metabolic pathway for their synthesis. The information available about them to date is summarized in reviews [24,25]. Our attention was focused on genetic and technological aspects of the production of the following antibiotics: mupirocin, gluconic acid, pyrrolnitrin (PRN), and 2,4-diacetylphloroglucinol (DAPG). All of the above antibiotics, except gluconic acid, are typical secondary metabolites, the production of which requires a separate genetic system—that is, an operon containing genes encoding the complete metabolic pathway. Gluconic acid is a product of primary metabolism, and numerous bacterial strains have the ability to synthesize it, but the key factor lies in the amount produced. Only those particular strains that generate sufficient quantities of gluconic acid to hinder the proliferation of other microbes can be identified as gluconic acid producers [26].

While the search for improved antibiotic producers from *Pseudomonas* strains is a common theme in the scientific literature, there is surprisingly limited discussion on their patenting and applications in biotechnology [27,28]. This gap in the research highlights the potential for innovative discoveries in this field [29].

The primary goal of this review was twofold. Firstly, to consolidate the information on the mechanisms of antibiotic synthesis by *Pseudomonas* strains and the control of these processes. The approaches for controlling antibiotic production were analyzed, encompassing genetic methods (such as mutagenesis of specific genes) as well as biochemical strategies, including modifying the cultivation medium composition and incorporating substances that may impact the final product yield.

The focus of the latter part of the study was on the implementation of antibiotic-producing *Pseudomonas* strains in the field of biotechnology. While traditional strains were commonly utilized, there were instances where genetically altered variants were employed. This study explored a range of techniques for modifying these producers in order to enhance the final product yield compared to the original strains.

Each section of this paper is focused on a separate antibiotic and contains information on the genetic organization of the biosynthesis pathway, the regulation of production, and the application of the antibiotic in medical and agricultural biotechnology.

Scientific information was searched using the Google and PubMed databases. More than 500 publications concerning antibiotic production by pseudomonads were analyzed. This review includes publications containing information on the mechanisms of biosynthesis, regulation and quantitative yields of the antibiotics. Patent searches were performed using the Google Patents, Justia Patents and PubChem services. The patent analysis focused on the efficiency of the antibiotic production and the method of obtaining the maximum yield.

## 2. Mupirocin

**Figure 1:**
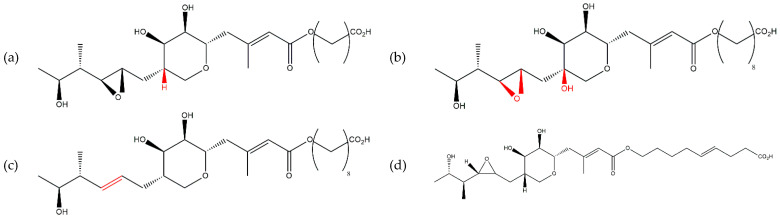
Chemical structures of pseudomonic acids (**a**) A, (**b**) B, (**c**) C, and (**d**) D.

Mupirocin is an antibiotic consisting of a mixture of pseudomonic acids (A, B, C, D) [30]. Mupirocin, also known as pseudomonic acids (Figure 1), is utilized for controlling skin infections and preventing postoperative wound inflammation when used intranasally [31,32].

**Figure 2:**
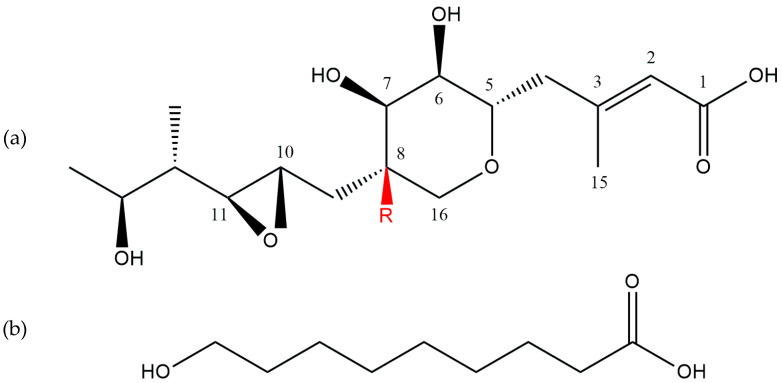
The main subunits of pseudomonic acids: (**a**) monic acid and (**b**) 9-hydroxynonanoic acid [30]. R = H or OH. All the structures were taken from PubChem, an open chemistry database.

The components of the mupirocin mixture have a similar structure and consist of two basic elements: monic acid and 9-hydroxynonanoic acid connected by an ester bond [32] (Figure 2).

Pseudomonic acid is produced by industrial strains of *P. fluorescens* as part of a mixture of metabolites. At least three variants of such mixtures are known (Table 1).

Since pseudomonic acid A was the dominant metabolite of the mixture (90–95%), it was it that exhibited the main antimicrobial effect on test cultures [37]. The other pseudomonic acids had the same spectrum of action, but their antimicrobial effect was weaker, possibly due to their lower quantity [36].

*Pseudomonas fluorescens* strain NCIMB 10586 is widely recognized as the leading producer of mupirocin [28,38,39]. Extensive research has focused on studying mupirocin production in this strain [40,41]. Besides *Pseudomonas fluorescens* NCIMB 10586, other pseudomonads belonging to the *P. fluorescens* species group have also been investigated for mupirocin production [28,42]. Haines et al. [43] demonstrated the close relationship between strain NCIMB 10586 and the species *P. synxantha* and *P. libaniensis*. Matthijs et al. [28] revealed that the closest relative of strain NCIMB 10586 is a type strain of the species *P. azotoformans*, with similarities also being observed with the type strains *P. cedrina* subsp. *cedrina* CFML 96-198T, *P. cedrina* subsp. *fulgida* DSM 14938T, *P. libanensis* CIP 105460T and *P. synxantha* IAM 12356. The intricate taxonomic relationships within the genus *Pseudomonas* make it challenging to establish clear boundaries between these species.

### 2.1. Mechanism of Mupirocin Biosynthesis

The pathway of mupirocin biosynthesis is regulated by a 74 kb gene cluster consisting of two transcriptional units, which can be roughly divided into two major modules [44,45] (Table 2).

A similar gene cluster was discovered in *Pseudomonas* sp. strain BRG100, which is utilized in agriculture for controlling *Setaria viridis* (Green Foxtail) and other pests [46,47]. A similar gene cluster but with a less resemblance was discovered by Haines et al. [43] in the genome of *Pseudomonas psychrotolerans* strain NS383.

The mutagenesis of individual elements of the mupirocin gene cluster showed the following:

1. The *mup*Q, *mup*S, *mup*T, and *mup*W genes were essential for the production of mupirocin, whereas *mup*O, *mup*U, *mup*V, and *macp*E were essential for the production of PA-A but not PA-B [45]. In this work, it was assumed for the first time that PA-B is a precursor of PA-A.
2. PA-C, previously assumed to be a precursor of PA-A, was formed by a minor parallel pathway [41]. Attempting to disable this pathway at the initial stage (Δ*mup*W) resulted in the loss of the ability to synthesize the major product (PA-A). Moreover, all the mutagenesis operations of the mupirocin cluster elements resulted in the loss of the ability to synthesize PA-A, but Δ*mup*C and Δ*mup*F retained PA-B production.

PA-C, the simplest component of the pseudomonic acid mixture, was chemically more stable than PA-A [41]. This was due to the presence of 10,11-epoxide in the structure of PA-A, which made it susceptible to intramolecular attack by the 7-OH. PA-C, which did not contain epoxide in its structure but had the same properties as PA-A and was more promising for pharmaceutical applications as an antibiotic. PA-C was produced by chemical synthesis [48,49,50,51], but attempts to obtain a high-yield producer of PA-C alone through modifications to the metabolic pathway have not been successful [41].

### 2.2. Regulation of Mupirocin Production

Two proteins encoded by the *mup*R and *mup*I genes governed the regulation of mupirocin production [52] (Table 3). Upon comparing their amino acid sequences with known sequences from the databases, it was revealed that *mup*R and *mup*I exhibit significant similarities to proteins associated with quorum-sensing regulation in *P. aeruginosa* and *Vibrio fischeri* [52].

The *mup* operon demonstrated peak expression during the stationary phase, while attempts to induce its expression in the exponential phase have proven unsuccessful, indicating active repression during this stage [52].

As mentioned earlier, mupirocin is produced as a mixture of the A, B, C, and D pseudomonic acids. According to the hypothesis, overexpression of *macp*E, *Mup*O, U, V, C, and F could increase the conversion of PA-B to PA-A, thus yielding a higher yield of the major metabolite [54]. However, Macioszek [53] and Gurney [55] showed that introducing these genes in trans into a wild-type strain did not increase the conversion of PA-B to PA-A, but it increased the yield of each of these compounds by two-fold proportionally.

Hothersall et al. [44] increased the efficiency of PA-A production through increased expression of the transcriptional regulator *Mup*R. The introduction of plasmids containing (1) *Mup*R only and (2) *Mup*R + *Mup*X into the wild-type strain (WT) increased the yield of PA-A by 4–5 times.

Genetic manipulation looks like a promising way to increase the proportion of pseudomonic acid A in the mixture and the overall production efficiency. However, we found no information on the patenting and use of mutant variants modified to produce only pseudomonic acid A. The only mention of such mutants was in the patent of Szell et al. [36] for *Pseudomonas* sp. strain No. 19/26 (NCAIM(P)B 001235). Basically, however, increasing the degree of production and the proportion of pseudomonic acid A in the mixture is currently achieved by modifying the cultivation conditions. In [56] the ratio of acids A and B in the mixture was 97:3, which was achieved by selecting the cultivation conditions of *Pseudomonas* sp. strain No. 19/26 (NCAIM(P)B 001235), namely pH 5.7, carbon source—dextrose, the concentration of which should be <0.5% for the entire period of production, addition of calcium chloride to a final concentration of 0.1% *w*/*w*. The authors obtained the maximum yield of pseudomonic acid A with 3.021 μg/g of culture medium, while the presence of pseudomonic acid in the product was 2.9% *w/w*.

### 2.3. Mupirocin in Biotechnology

One of its advantages compared to other antibiotics is its low toxicity to humans and animals [36]. Primarily, it was employed against *Staphylococcus aureus*, but it also demonstrated effectiveness against *Streptococcus* [57], *Neisseria* [58], as well as several other Gram-positive and Gram-negative bacteria [59,60,61,62].

The best-known mupirocin-based preparations are Bactroban (UK), Turixin (Germany) and Bactoderm (Israel) [40]. These preparations are available in the form of creams and ointments containing 2% of the active ingredient. Bactroban ointment, nasal agent and cream are protected by patents US4,524,075 [63], US4,790,989 [64] and US6,025,389 [65], respectively. The ointment contains mupirocin, while the nasal agent and cream contain crystalline mupirocin calcium dihydrate [66].

The original mupirocin patent [34] detailed the process of creating the sodium variant of pseudomonate. However, due to its hygroscopic nature, Curzons proposed the utilization of lithium pseudomonate instead [67,68]. Lithium pseudomonate proved to be a reliable and non-hygroscopic crystalline compound, ensuring its stability over time. The works [53,67] used the same producer, *Pseudomonas fluorescens* strain NCIB 10586. Lithium pseudomonate (mupirocin lithium) is available as an analytical preparation from Sigma (Burlington, MA, USA) or Thermo Fisher Scientific (Waltham, MA, USA), but it is not used for drug production, unlike mupirocin calcium. The calcium salt of mupirocin is used in ointments, as is pseudomonic acid.

Patents for crystalline [69,70,71,72] and amorphous [73] salts of pseudomonic acid are known. Previously, it has been repeatedly argued that amorphous calcium pseudomonate cannot withstand high temperatures and degrades during storage [69,71,74], whereas the crystalline form is convenient for pharmaceutical use. However, Weisman et al. [73] showed that in terms of the temperature stability, amorphous calcium pseudomonate is comparable to pseudomonic acid, which is widely used in pharmaceuticals. Calcium pseudomonate is available as an analytical preparation from Sigma.

## 3. Gluconic Acid

**Figure 3:**
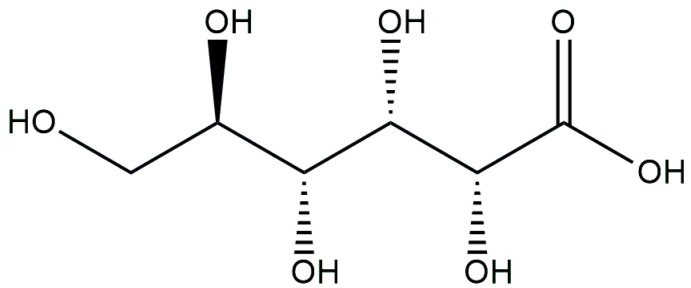
Chemical structure of gluconic acid.

Gluconic acid (Figure 3) possesses antifungal properties and is commonly employed to control plant diseases [75,76,77,78,79]. Gram-negative bacteria utilize the oxidation of glucose to gluconic acid, which takes place in the bacterial periplasm, as a means of solubilizing inorganic phosphorus. This process provides them with a competitive advantage over other organisms, as it converts accessible carbon sources into ones that are less available to other microorganisms [80,81].

The first *Pseudomonas* strains in which gluconic acid production was observed were *Pseudomonas ovalis* NRRL B1486 [82,83,84] and *P. ovalis* NRRL B-SS [75] obtained from the US Dept of Agriculture, Peoria, USA. Gluconic acid was the major organic acid produced during phosphate solubilization in *Pseudomonas* sp. [85], *P. fluorescens* [86], *P. savastanoi* [87] and *Pseudomonas corrugata* [88].

### 3.1. Gluconic Acid Biosynthesis Pathway

The pathway of gluconic acid biosynthesis can be categorized as a fundamental metabolic process. Unlike fungi, where the conversion of glucose to gluconic acid is regulated by glucose oxidase (b-D-glucose: oxygen 1-oxidoreductase, E.C. 1.1.3.4) [89,90], bacteria employ glucose dehydrogenase (GDH, E.C. 1.1.99.17) to perform this reaction [91,92,93]. The glucose dehydrogenase enzyme is situated in the membrane and is induced by high concentrations of glucose (>15 mM) [94]. While gluconic acid participates in the bacterial cell’s pentose phosphate pathway, its production is repressed at elevated (>15 mM) glucose levels, leading to the accumulation of this metabolite in the environment [87].

Currently, the best-known producer of gluconic acid is the *Pseudomonas* strain AN5 [26,95,96]. The production of other antibiotics common for *Pseudomonas* strains (phenazine-1-carboxylic acid (PCA) and DAPG) was absent in strain AN5 [78].

### 3.2. Regulation of Gluconic Acid Production

Based on *Pseudomonas* AN5, two transposon mutants (AN5MN1 and AN5MN2) with no antimicrobial properties have been constructed [26]. Yu et al. [79], also through transposon mutagenesis of the strain *Pseudomonas capeferrum* WCS358, showed that two genes are required for gluconic acid biosynthesis: *pqq*F and *cyo*B. *Pqq*F was a putative protease involved in pyrroloquinoline quinone (PQQ) biosynthesis [97,98]. PQQ functioned as a cofactor in many carbon utilization reactions [99]. In turn, CyoB was a subunit of the terminal ubiquinol cytochrome bo3 oxidase (CYO) complex, which was required in the aerobic respiratory chain for energy generation [100]. Both mutant variants demonstrated a reduced ability to produce organic acids, and therefore, reduced the environmental pH less efficiently than the wild-type strain [79].

Galet et al. [77] conducted Tn5 mutagenesis and found that the *pqq*E gene plays a critical role in the biosynthesis of gluconic acid. A mutant variant of *P. fluorescens* strain BBc6R8 with a defective *pqq*E gene was incapable of gluconic acid production, and a similar mutant of *P. fluorescens* strain F113 produced very small amounts of organic acid [101]. Galet et al. [77] suggested that the pQQFABCDE operon was required for gluconic acid biosynthesis in pseudomonads.

Trivedi and Sa [88] conducted a genetic modification experiment on a *Pseudomonas corrugata* strain (NRRL B-30409) using N’-methyl-N’-nitro-N-nitrosoguanidine (NTG), aiming to create cold-tolerant mutants capable of efficiently solubilizing phosphate at lower temperatures. Nevertheless, it is important to note that while chemical-induced mutagenesis can result in enhanced production efficiency in terms of the desired compound, it does not ensure the reproducibility of the experiment and thus cannot be considered a reliable approach for the development of biotechnologically promising producers [102].

De Werra et al. [76] investigated the engineering of *Pseudomonas fluorescens* strain CHA0 to enhance the production of gluconic acid [76]. They found that by disabling the *gad* (gluconate dehydrogenase) gene, which is responsible for the conversion reaction of gluconic acid to 2-ketogluconate, the cells accumulated a higher concentration of gluconic acid compared to the wild-type strain. Interestingly, the mutant variants of the *gcd* and *gad* genes exhibited different capabilities in terms of producing other antifungal components, namely DAPG and pyoluteorin (PLT). The mutant strain Δ*gcd,* with glucose dehydrogenase inactivation, demonstrated a more efficient production of DAPG compared to the wild-type strain. Additionally, the Δ*gcd* mutant produced 10 times more DAPG than the Δ*gad* mutant (4 μM) after just 1 day of cultivation. Moreover, the Δ*gcd* mutant showed the ability to produce PLT, while this component was absent in the wild-type strain. Thus, it could be concluded that the Δ*gcd* mutant was more effective in producing the antifungal components DAPG and PLT, while the Δ*gad* mutant was more efficient as a producer of gluconic acid.

In addition to the antifungal properties, *Pseudomonas* strains producing gluconic acid can participate in intermicrobial interactions in open ecosystems. Decreasing the pH of the cultivation medium due to gluconic acid production by *P. fluorescens* strain BBc6R8 in [77] prevented the production of γ-actinorhodin by *S. coelicolor*. Actinorhodin is a benzoisochromanequinone dimer polyketide antibiotic whose production is characteristic of *Streptomyces* [103,104]. All the gluconic acid producers used by Galet et al. [77] (*P. fluorescens* SBW25 [105], *P. fluorescens* Pf0-1 [106], *P. protegens* Pf-5 [107] and *P. aeruginosa* PAO1 [108]) showed similar effects on actinorhodin production by *Streptomyces* strains. The demonstrated effect was not due to specific properties of gluconic acid but to a general decrease in the pH of the cultivation medium.

In addition to *Streptomyces*, the effect of gluconic acid on the production of the siderophore pyoverdine by *P. putida* is also known. Ponraj et al. [109] showed that when the pH of the medium decreases, the efficiency of the pyoverdine production decreases, which may ultimately affect the efficiency of the plant root colonization. Ponraj et al. [109] noted that in the case of pyoverdine, there was no effect of gluconic acid on the expression of biosynthetic genes. A decrease in the pH of the medium affects the periplasmic transformation of pyoverdine and its secretion from the cell.

### 3.3. Gluconic Acid in Biotechnology

As the subject of patents in the USA and Europe [26,78], strain AN5 has emerged as a significant producer of gluconic acid. Its original purpose was to combat *Gaeumannomyces graminis var. tritici* (Ggt), but subsequent findings [78] revealed that the gluconic acid derived from strain AN5 possessed valuable properties against *Deuteromycetes* (*E. pupurescens*, *Alternaria* sp., *A. oligosporus*, *M. fructocola*, *B. cinerea*, *V. dahlia*), *Basidiomycetes* (*F. annosus*, *A. mellea*, *B. granulatus*, *P. suphureus*), *S. cerevisiae*, as well as a broad spectrum of both Gram-negative and Gram-positive bacteria.

According to the research, *Pseudomonas* strain AN5 exhibited a gluconic acid production rate of approximately 5.0 mg/mL. Notably, Kaur et al. [26] emphasized that there were no known reports of other pseudomonads with similar levels of gluconic acid production. Nevertheless, Vyas and Gulati [110] observed in *P. trivialis*, *P. poae* and *P. fluorescens* strains the production of gluconic acid at approximately 17,000–19,000 μg/mL (17–19 mg/mL) during tricalcium phosphate solubilization.

## 4. PRN

**Figure 4:**
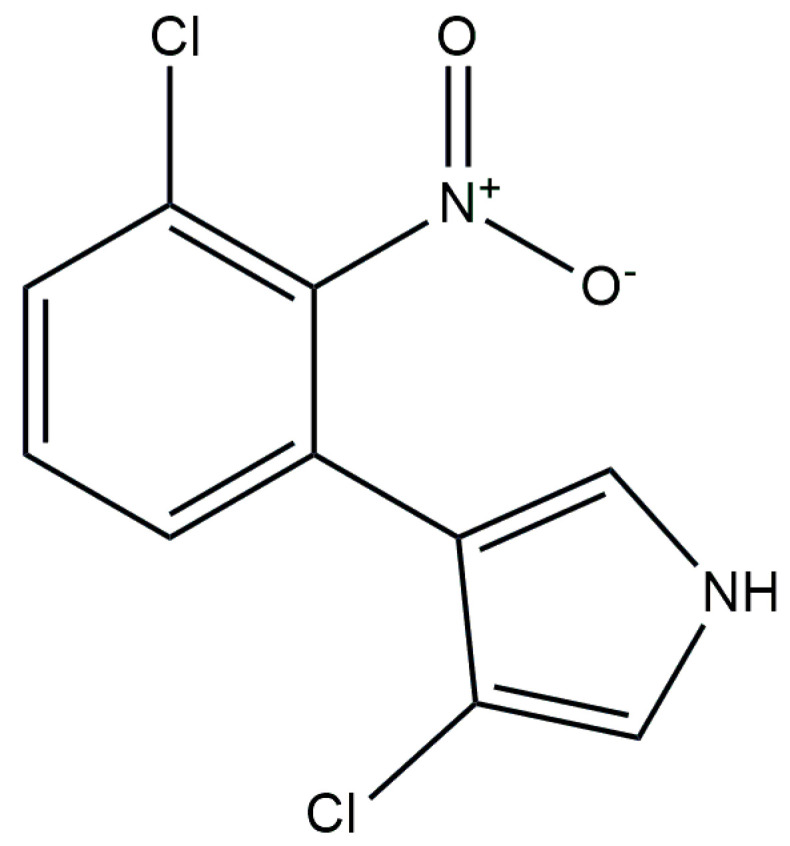
Chemical structure of PRN.

PRN [3-chloro-4-(2-nitro-3-chlorophenyl)-pyrrole] (Figure 4) exhibited notable antifungal properties, effectively combating a wide range of fungal infections (*Alternaria* sp., *Botrytis cinerea*, *Pythium aphanidermatum*, *P. ultimum*, *Rhizoctonia solani*, *Rhizopus* sp., *Aspergillus niger*, *Fusarium oxysporum*, *Penicillium expansum*, and *Sclerotium rolfsii*) and bacteria (*Agrobacterium tumefaciens*, *Corynebacterium insidiousum*, *Pseudomonas syringae* pv. *syringae*, and *Xanthomonas campestris*) [111]. It was first isolated from *Pseudomonas pyrrocinia* [112] in 1964. PRN producers belonging to different *Pseudomonas* species are now known: *P. fluorescens* [113,114,115], *P. chlororaphis* [116,117,118,119], and *P. cepacia* [120]. PRN is composed of a benzene ring and a pyrrole ring, each adorned with chlorine atoms. Additionally, the benzene ring harbors a nitro group.

### 4.1. Mechanism of PRN Biosynthesis

**Figure 5:**
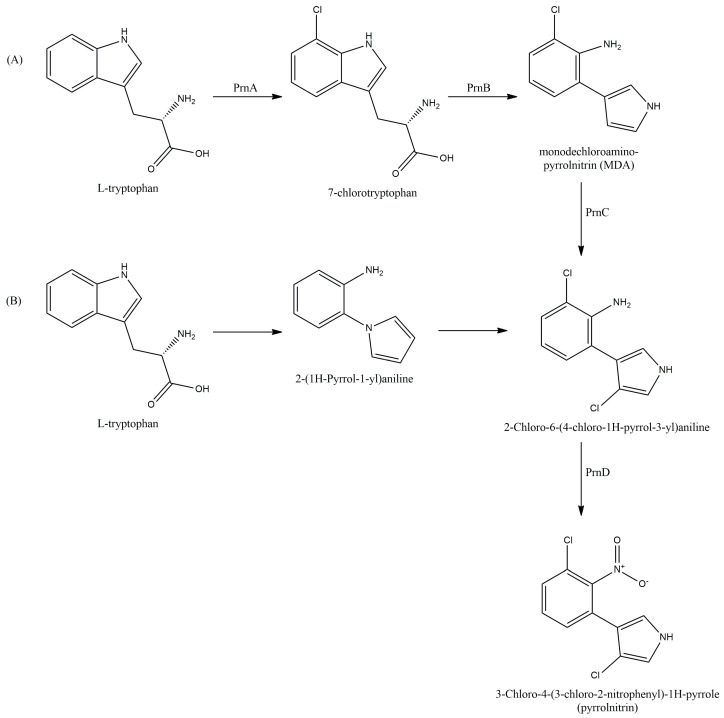
PRN biosynthesis pathway according to (**A**) van Pée et al. [125] and (**B**) Chang et al. [126].

Tryptophan serves as a direct precursor of PRN, with both L- [121] and D- [122] forms being available. Microorganisms could utilize tryptophan analogs introduced into the culture medium to synthesize PRN-like compounds. However, it should be noted that these compounds exhibited lower antimicrobial activity compared to PRN [122,123]. The synthesis of PRN from tryptophan involved a series of four reactions regulated by the *prn* operon (Figure 5) [65,113,116,123,124].

Hammer et al. [113] discovered a 5.8 kb operon containing four genes responsible for PRN biosynthesis in the genome of a *P. fluorescens* strain. The authors named this operon *prn*A-D. However, Kirner et al. [115] and van Pée [127] later revealed that chloroperoxidases, previously believed to be involved in PRN biosynthesis [128], were not actually involved in this process. Using the *prn* operon genes as a reference for cloning and sequencing homologous genes, Hammer et al. [116] demonstrated that the *prn* gene cluster was highly conserved and could be found in other *Pseudomonas* strains, *Burkholderia cepacia* and *Myxococcus fulvus*.

PrnA and PrnB proteins have been isolated and thoroughly characterized [124,129], while *prn*D has been 3-D modeled to determine its protein structure [130]. De Souza et al. [131] developed a method to detect *prn*D in soils to assess the potential diversity of producers in ecosystems.

In contrast to previous findings by van Pée regarding the *prn* metabolic pathway [125], Chang et al. [126] proposed that the product of the first reaction of the PRN biosynthesis pathway in *P. aureofaciens* may be aminophenylpyrrole rather than 7-chlorotryptophan.

### 4.2. Regulation of PRN Production

The synthesis of numerous secondary metabolites is regulated by global regulators. For example, mutations in the *gac*A gene in *P. fluorescens* strain CHA0 [132] impede the production of DAPG, hydrogen cyanide (HCN), and pyoluteorin. Similarly, in the *Pseudomonas fluorescens* strain Pf-5 that failed to produce PRN, a mutation was detected in the *rpo*S gene, the product of which was involved in the biosynthesis of secondary metabolites, particularly PRN [133,134]. By introducing a plasmid containing a functional copy of *rpo*S, the researchers managed to restore the PRN production ability in *Pseudomonas fluorescens* strain Pf-5 [133]. In a study conducted by Park et al. [119] using the *P. chlororaphis* strain, it was demonstrated that functional (not defective) *rpo*S, *gac*S (a component of the two-component GacS/GacA system), and *prn*A genes are necessary for PRN production. The highest expression level of *prn*A was observed during the stationary phase of growth.

In contrast with Saringuet et al. [133], Manuel et al. [135] showed that a mutation in the *rpo*S gene of *Pseudomonas chlororaphis* strain PA23 conferred the increased antifungal properties of the strain against *Sclerotinia sclerotiorum* and elevated the PRN, lipase and protease production. When *rpo*S was added in trans, the production levels of the above compounds returned to those of the wild-type strain. Given the difference in the effect of mutant *rpo*S on PRN production by *Pseudomonas fluorescens* Pf-5 [133] and *Pseudomonas chlororaphis* PA23 strains (negative in the former and positive in the latter), Manuel et al. [135] suggested that the difference may be due to the different spectrum of metabolites produced by the strains, or that RpoS may affect PRN expression not directly but through regulatory elements different in these bacteria.

Another regulator of PRN biosynthesis is the *vfr* gene [136]. Vfr is a cAMP-dependent global regulator of virulence factor expression in *Pseudomonas* [137,138,139]. By knockout mutagenesis, it was shown that when the *vfr* gene is switched off, the *prn* operon expression critically drops and the amount of PRN produced is quite negligible. At the same time, the level of PCA production was unchanged in *vfr*-defective variants. Wu et al. [136] concluded that Vfr is a regulator of only PRN production, not of phenazines.

Huang et al. [117] used knockout mutant variants of *Pseudomonas chlororaphis* strain G05 (Δ*phz*, Δ*prn* and Δ*phz*Δ*prn*) to prove that disabling the phenazine biosynthesis cluster does not affect the antifungal capabilities of the strain too much. This work showed for the first time that it is not phenazines but PRN that plays a key role in the control of *F. graminearum* and the bioprotection of cereal crops against diseases.

Park et al. [119] showed that the presence of glucose in the culture medium inhibited the production of PRN by the *Pseudomonas chlororaphis* strain. On medium without glucose, the product yield was 1.7 µg/mL, while when glucose was added, the yield was 0.2 µg/mL. Interestingly, the effect on PCA production was reversed: 2.5 µg/mL without glucose and 27.4 µg/mL with glucose. In this work, as well as in [117], it was demonstrated that the antifungal effect on *F. graminearum* and *R. solani* is exerted more by PRN than by phenazines.

Keum et al. [140] demonstrated that the production of PRN by pseudomonads was influenced by the presence of xenobiotics, particularly pesticides. In the case of *Pseudomonas fluorescens* strain Pf-5, accumulation of PRN (up to 6 mg/L) or amino-PRN (up to 7 mg/L), respectively, was observed when cultured in the presence of 10 μm fenpiclonil or fludioxonil. Additionally, when cultured with fludioxonil, the culture medium contained approximately 2.5 mg/L of PRN. As for the control group without pesticides, the accumulation of approximately 2 mg/L PRN and 7.5 mg/L amino-PRN was observed. Thus, fenpiclonil was found to increase the production of PRN by three times, while fludioxonil did not affect the yield of PRN but influenced the production of the intermediate metabolite, amino-PRN.

### 4.3. Modifications of PRN Producers

The first attempt to modify a PRN producer was made by Salcher and Lingens in 1979 [141]. They conducted a study to enhance the production efficiency of PRN production in *Pseudomonas aureofaciens* strain ATCC 15926 using variations in the culture conditions [142] and chemical mutagenesis. The original strain yielded 0.3 μg/mL PRN, and efforts to increase the yield through media selection proved futile. To induce mutations, the authors employed N-methyl-N′-nitro-N-nitrosoguanidine (MNNG) as a mutagen. Remarkably, the *P. aureofaciens* ACN mutant variant exhibited the best outcome, producing 9 μg/mL (9 mg/L) of PRN. Notably, the ACN variant colonies lacked color, contrary to the orange colonies of the parental strain. However, research by Salcher and Lingens in 1979 [141] revealed that both strains produced PCA, and the lack of color in the mutant may be due to an inability to synthesize 2-hydroxyphenazine-1-carboxylic acid [143]. Encouragingly, the ACN mutant variant remained stable over an extended period without reverting to the wild-type state.

In a groundbreaking study, Zhang et al. [144] successfully transferred the *prn* operon from *P. protegens* (formerly *P. fluorescens* [145]) Pf-5 into *P. synxantha* 2-79. The recipient strain initially possessed the ability to produce phenazines and displayed effectiveness against both take-all and *Rhizoctonia* root rot of wheat. This remarkable research marked the first documented instance where a *prn* operon introduced through transfer into a phenazine-producing recipient strain demonstrated consistent maintenance and expression. Moreover, the resulting recombinants exhibited significantly enhanced biocontrol properties when compared to the wild-type strain. Of notable importance, these recombinants displayed remarkable effectiveness against various pathogens, including *Rhizoctonia solani*, *Gaeumannomyces graminis* var. *tritici*, *Sclerotinia sclerotiorum*, *Fusarium culmorum*, and *F. pseudograminearum* significantly, surpassing the capabilities of *P. synxantha* 2-79.

### 4.4. PRN in Biotechnology

A series of *P. fluorescens* strains producing PRN have been patented in the USA for the purpose of managing diseases caused by *Rhizoctonia* and *Pythium* [146]. Additionally, *P. cepacia*, which has been isolated from the surface of apple leaves, has been patented as a method to control postharvest diseases in pome fruits [147]. While PRN is the only antibiotic produced by the aforementioned strains in the examples provided, there are instances where PRN was produced in conjunction with other compounds. *Pseudomonas* sp. strain DSM21663 [148] served as a producer of PRN, DAPG, and indole acetic acid. The aim of the patent was to utilize this particular strain for the management of the soil-borne root and foliar pathogens originating from both fungal and bacterial sources. Strain DSM21663 was classified as a fluorescent pseudomonad, with the 16S rRNA gene sequence demonstrating a similarity of 98.6% with *P. congelans* and 97.3% similarity with *P. fluorescens*.

Commercial preparations utilizing PRN derivatives (fludioxonil and fenpiclonil) have been registered in France and Switzerland (Table 4). The drugs are intended for use as fungicides and in agriculture [123].

The products have a long history of use (since 1993), since they are not microbial preparations but combinations of chemical compounds. No commercial bacterial preparations where PRN-producing pseudomonads have been used for plant defense have been found. Purified (≥98% purity) PRN from *P. cepacia* is sold by Sigma as a standard in the detection of PRN in *Serratia marcescens* cell culture extract [149].

## 5. DAPG

**Figure 6:**
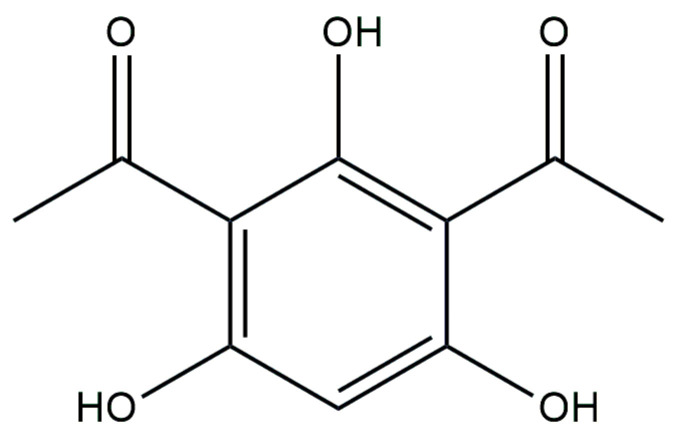
Chemical structure of 1,1′-(2,4,6-Trihydroxy-1,3-phenylene)di(ethan-1-one) (DAPG).

DAPG, as shown in Figure 6, is a polyketide antibiotic. It was previously believed that its production was specific to bacteria of the *P. fluorescens* species group (including *P. protegens*, *P. brassicacearum*, *P. kilonensis*, and *P. thivervalensis*). The genes responsible for DAPG synthesis were thought to have a monophyletic distribution, meaning they originated from a common ancestor [150]. However, a recent study by Almario et al. [151], which involved analyzing the genomes of various *Pseudomonas* strains, challenged this view. The researchers discovered that the genes involved in the biosynthesis pathway of DAPG biosynthesis (*phl*) were acquired independently through multiple events rather than being inherited vertically from a single ancestor. Almario et al. [151] found *phl* genes in pseudomonads, beyond *P. fluorescens*, as well as in two genera of *Betaproteobacteria*.

In 1993, using the example of the strain *Pseudomonas* sp. F113, it was shown that the precursor of DAPG is MAPG, and an acyltransferase responsible for the conversion of MAPG to DAPG was described [152]. The genes for the DAPG biosynthesis pathway were first identified by Bangera and Thomashow [153] using *Pseudomonas fluorescens* strain Q2-87 as an example. The DAPG biosynthesis pathway has now been described in detail [151,154].

*P. fluorescens* strain Pf-5, originally identified as the best-known DAPG producer [155], was later reidentified as *P. protegens* [156]. Nowak-Thompson et al. [155] revealed that the composition and quantity of compounds produced by the strain (DAPG/pyoluteorin/PRN), as well as the absolute amount of each, varies depending on the culture medium. In particular, when 2% glucose was added as a carbon source, DAPG was the primary compound produced and was synthesized at 16.6 mg/L. However, when glucose was replaced by glycerol, the amount of synthesized DAPG dropped sharply (to 0.19 mg/L), but pyoluteorin was synthesized in the amount of 5.75 mg/L. These findings obtained by Nowak-Thompson et al. [155] for *P. fluorescens (protegens)* Pf-5 contrast with those obtained earlier by Shanahan et al. [157] for *Pseudomonas* sp. F113. The strain *Pseudomonas* sp. F113 actively produced DAPG when growing on sucrose, mannitol, and fructose, but no DAPG synthesis occurred on glucose despite the high numbers of growing cells.

### 5.1. Mechanism of DAPG Biosynthesis

Bangera and Thomashow [153] identified a 6.5 kb region consisting of six reading frames in strain Q2-87. Five of these (*phl*A, *phl*C, *phl*B, *phl*D, and *phl*E) were transcribed in one direction, *phl*F in the opposite direction. The authors demonstrated through knockout mutagenesis of the region components that (1) *phl*D was required for MAPG synthesis, and the mutant strain with a defective *phl*D gene was able to convert MAPG to DAPG but not to perform MAPG synthesis, and (2) *phl*A, B and C were required for MAPG to DAPG conversion. The protein products of these genes acted as a single complex: knockout of A, B, or C, even with functional D, resulted in the inability to synthesize MAPG and DAPG. Bangera and Thomashow [153] initially proposed that the reaction controlled by *phl*D used acetoacetylCoA as the starting unit, yielding MAPG. However, later, Achkar et al. [158] discovered that this reaction involves the formation of phloroglucinol from malonylCoA. It is suggested that *phl*D is responsible for the formation and cyclization of an activated 3,5,7-triketooctanoate [153,158] to synthesize MAPG, which is then acetylated to provide DAPG. Bangera and Thomashow [153] noted the relatedness of *phl*D to plant chalcone/stilbene synthases (CHS/STS). This is unusual, since antibiotic biosynthesis by bacteria is mainly controlled by type I and type II polyketide synthases [155,159,160]. Furthermore, Gupta et al. [161] confirmed the relationship between the *phl* operon and the CHS/STS plant gene family by observing the absence of the acyl carrier protein gene.

Inactivation of *phl*A [162,163] and *phl*C [164] resulted in loss of the ability to produce DAPG. Generally, at the sequence level, components of the DAPG operon were conserved within the genus *Pseudomonas* [165]. However, Dash et al. [165], in their study of a strain of *P. fluorescens* from India, identified a unique *phl*B gene in the strain that differs in the nucleotide sequence from the *phl*B of other pseudomonads (2P24 [166], P12 [151] and Q37-87 [167,168]). Interestingly, the *phl*B strain from Dash et al.’s study [165] showed high affinity with the *phl*B strain of *Pseudomonas fluorescens* Q2-87 [153] in terms of the amino acid sequences.

Bangera and Thomashow [153] suggested that *phl*A, *phl*C and *phl*B have dual roles: they are required for the conversion of MAPG to DAPG and they participate in MAPG biosynthesis.

The *phl* operon is flanked downstream by *phl*E and upstream by *phl*F, *phl*G, and *phl*H genes [169]. The *phl*E gene product is responsible for the release of DAPG outside the cell [153,170]. More recently, the role of *phl*E as a permease was confirmed by Abbas et al. [171] in *P. fluorescens* strain F113. They also suggested that the *phl*E gene product was involved in the resistance of the cells themselves to DAPG. A mutant variant of strain F113 defective in the *phl*E gene showed greater sensitivity to osmotic, oxidative and heat-shock stresses. The authors also noted that *phl*E knockout led to a decrease in DAPG production because the cells became sensitive to extracellular DAPG.

PhlG was a crucial hydrolase responsible for the degradation of DAPG back to MAPG [172,173]. This process played a vital role in controlling the extracellular DAPG levels. Excessive amounts of DAPG were toxic to the cells, so when its concentration exceeded the acceptable level, a process was triggered to convert DAPG into the less toxic MAPG [174] by cleavage of a carbon–carbon bond linking an acetyl group to the phenolic ring [175]. Zhao et al. [174] demonstrated that the deletion of *phl*G in *Pseudomonas fluorescens* strain 2P24 did not affect its antifungal activity and ability to colonize plants. The authors noted that the opposite would be logical, since a variant strain with defective *phl*G should have more DAPG. The structure of the PhlG protein and its catalytic features have been extensively described by Saitou et al. [176] and He et al. [175].

Both *phl*F and *phl*H are *tet*R-like repressors. PhlF inhibited transcription of the *phl* operon by binding to the inverted *ph*O repeat, which was located downstream of the *phl*A transcriptional start site [177,178]. *Phl*H inhibited transcription of the *phl*G gene by binding to a motif overlapping with the −35 site recognized by σ70 factors [173,179,180].

There is also *phl*I, but its function is still unknown [181]. Yu et al. [182] studied the effect of flavonoids on DAPG production by *Pseudomonas fluorescens* strain 2P24 and found that exposure to apigenin and phloretin resulted in decreased expression of the *phl* operon, including *phl*E. At the same time, there was no effect of apigenin and phloretin on the expression of *phl*I, which the authors suggested is cotranscribed with *phl*E. This suggested that *phl*I was not directly involved in DAPG biosynthesis [182].

### 5.2. Regulation of DAPG Biosynthesis

DAPG biosynthesis in pseudomonads is regulated by a number of factors:

1. By the TetR family transcriptional repressors *phl*F and *phl*H [173,177,183].

2. At the post-transcriptional level using the Gac/Rsm system [174,184].

Both of these mechanisms, as well as the co-regulation of DAPG and pioluteorin production, are described in detail in a review [185]. We will consider factors that are of a different nature.

3. *Psr*A

Similar to *phl*F, *psr*A is a transcriptional repressor that affects the DAPG biosynthesis genes by directly binding to a binding box localized on the *phl*A promoter region. Wu et al. [178], using *P. fluorescens* strain 2P24 as an example, revealed why two regulators, seemingly identical in their mechanism of action, are maintained in the genome. The *phl*F- mutant produced approximately 20-fold more DAPG than the *psr*A mutant; hence, regulation by *phl*F is dominant for DAPG production [178]. *Psr*A affected DAPG biosynthesis by activating the expression of the *rsm*A gene through the sigma factor RpoS.

4. *Opr*F/*Sig*X

Bangera and Thomashow [153] demonstrated that a *Pseudomonas* strain, which had a transposon insertion in the *opr*F gene, exhibited an increased quantity of red pigment of an unknown nature in comparison to the original strain. In *P. fluorescens* strain 2P24, the *sig*X gene was found to be positioned upstream of the *opr*F gene and acted as a positive regulator of *opr*F. Li et al. [186] showed that *sig*X did not affect the expression of the *phl* operon components but intensified the expression of the acetyl CoA carboxylases involved in malonyl-CoA synthesis. Malonyl-CoA served as the initial substrate in the DAPG biosynthesis pathway. The authors also revealed that the regulation of DAPG production by *opr*F is *sig*X-dependent and this process is unrelated to the Gac/Rsm system. Furthermore, the transcription of *sig*X was intensified by salt starvation or the addition of glycine to the medium [186].

5. *Hfq*

In their study, Xiao-Gang et al. [187] utilized transposon mutagenesis to discover yet another key regulator of the production of DAPG in *P. fluorescens* strain 2P24, the *hfq* gene. Inserting the transposon into the *hfq* gene led to a noteworthy decrease in *phl*A expression and the amount of DAPG in the medium. Furthermore, the *hfq* gene was revealed to play a vital role in the expression of *pco*I synthase, a component of the quorum-sensing system in pseudomonads. As a consequence of *hfq* inactivation, not only was DAPG production affected, but the ability to establish biofilms and successfully colonize plants was also significantly diminished.

6. GrxD

Dong et al. [188] showed the effect of the monothiol glutaredoxin GrxD on DAPG production by *P. fluorescens* strain 2P24. The level of DAPG production was dramatically decreased when *grx*D was inactivated. Furthermore, site-directed mutagenesis of the CGFS motif in GrxD demonstrated that this motif is directly involved in the regulation of DAPG synthesis. The authors hypothesized that the positive regulation of DAPG production by GrxD was mediated by the Gac/Rsm signaling cascade. In addition, GrxD negatively affected the expression of *phl*F, which intensified the DAPG production in cells.

7. DsbA1

Zhang et al. [154] demonstrated the negative regulation of DAPG production by the disulfide oxidoreductase *dsb*A1. DsbA1 affected DAPG production not through the Gac/Rsm system but through the membrane-bound glucose dehydrogenase *gcd*. DsbA1 interacted with Gcd, which modulated the DAPG production. Site-directed mutagenesis of the cysteine residues in DsbA1 resulted in increased Gcd activity and intensified DAPG production.

When the *gcd* gene was deactivated, it led to a significant reduction of approximately 60% in DAPG production. However, in a study conducted by De Werra et al. [76], mutagenesis of *gcd* in *P. protegens* strain CHA0 resulted in the opposite outcome, with a strong accumulation of DAPG in the culture medium. Zhang et al. [154] suggested that the possible reasons for this were (1) the difference in the mechanisms of glucose catabolism in the strains, and (2) the different spectrum of antibiotics produced by the strains. Here, 2P24 produced only DAPG, while CHA0 produced DAPG, pyoluteorin (Plt), and PRN.

8. RetS

RetS is a regulator of exopolysaccharide and type III secretion [189]. Liu et al. [190] showed that RetS regulates DAPG synthesis through the Gac/Rsm signaling cascade, and the inactivation of RetS leads to increased production of DAPG and a red pigment of an unknown nature, which was also detected by Bangera and Thomashow [153] during mutagenesis of *P. fluorescens* strain 2P24 by the *opr*F gene.

He et al. [191] reported two additional genes in *P. fluorescens* strain HP72 that are involved in the regulation of DAPG biosynthesis. The authors designated them as ORFA and ORFB and showed that their inactivation resulted in the inability of the strain to synthesize DAPG. ORFA was related to a putative transport protein from *P. putida*, and ORFB—to a GNTR-like regulator (possibly *phl*H).

### 5.3. Producer Modifications

A study conducted by Zhou et al. [192] utilized *Pseudomonas fluorescens* strain J2 as a model to investigate the impact of *phl*F gene inactivation on DAPG production of almost 3-fold relative to the parental strain (3.14 mg/L vs. 1.12 mg/L). Despite this significant increase, the growth characteristics of the cultures remained largely unchanged. Similar results were obtained by Schnider-Keel et al. [180] for *P. fluorescens* strain CHA0, in which the amount of DAPG produced increased 4-fold upon *phl*F inactivation. Furthermore, Wu et al. [178] observed that *P. fluorescens* strain 2P24 with an inactivated *phl*F gene produced 5 mg/L DAPG (vs. 0.05 mg/L in the parental strain), i.e., the authors demonstrated a 100-fold enhancement of production. Notably, the authors also discovered that overexpression of *psr*A in inactivated *phl*F results in a 2-fold decrease in DAPG.

At the same time, in their study, Delany et al. [183] observed that inactivating *phl*F in *Pseudomonas fluorescens* strain F113 resulted in reduced DAPG production compared to only the parental strain during the initial stages of culture growth. DAPG was present in the mutant culture medium as early as 6 h of growth, whereas in the parental strain appeared at 12 h. The authors explained the slower growth rate of the mutant strain by the early presence of DAPG in the medium at the early stages of culture growth, and they also noted that both cultures had similar amounts of DAPG in the medium by the end of the exponential phase.

Further experimentation with *Pseudomonas fluorescens* strain F113 revealed that overexpression of the *prr*B gene encoding a regulatory RNA molecule increased the production of DAPG in the parental strain and also restored the ability to produce DAPG in mutant variants defective in the *gac*S (formerly *lem*A) and *gac*A genes [177,193].

Wu et al. [178] revealed that the *psr*A gene functions in regulating DAPG production in the strain *P. fluorescens* strain 2P24. PsrA bound to an operator in the promoter region of *phl*A and thus negatively controlled *phl*A function at the transcriptional level. In addition, PsrA affected the expression of RpoS and RsmA at the post-transcriptional level. Inactivation of the *psr*A gene resulted in a 5-fold increase in the amount of DAPG produced (250 mg/L vs. 50 mg/L). The *rpo*S mutant of the same strain was able to produce ~3-fold more DAPG than the parental strain (~175 mg/L vs. 50 mg/L). The data obtained were consistent with the experimental materials of Sarniguet et al. [133] for the *P. protegens* strain Pf-5. The parental strain *P. protegens* Pf-5 produced PRN (2.2 mg/L), pioluteorin (6.6 mg/L), and negligible (<0.1 mg/L) amounts of DAPG. The *rpo*S mutant variant lost the ability to produce PRN, but pyoluteorin was synthesized in amounts of 37.7 mg/L and DAPG in amounts of 52.2 mg/L.

Mutagenesis of the *ret*S gene in *P. protegens* strain Pf-5 [156] resulted in a 20–30-fold increase in the amount of DAPG produced.

Achkar et al. [158] conducted a study to investigate the enhanced production of phloroglucinols (phloroglucinol, MAPG and DAPG) in the *P. protegens* strain Pf-5 transformed with the integration of the plasmid pJA2.232. The plasmid pJA2.232 was derived from pME6031 [194] and contained the *phl* operon as an additional component. The objective of introducing another copy of the *phl* operon into the strain was to bypass the regulation of DAPG synthesis by *phl*F. Consequently, the levels of MAPG and DAPG synthesis rose approximately 20-fold compared to the control, while phloroglucinol saw a remarkable 47-fold increase. The parental strain yielded 35 mg/L of DAPG, whereas the variant with PJA2.232 produced a significantly higher amount of 790 mg/L.

Mutagenesis of the disulfide oxidoreductase *dsb*A1 gene plays a crucial role in regulating DAPG production by fine-tuning the function of glucose dehydrogenase (Gcd). A recent study [154] revealed that the overexpression of the *dsb*A1 gene led to a significant increase in DAPG production in *P. fluorescens* 2P24, approximately doubling the amount from 10 mg/L to around 18 mg/L. Additionally, the authors also performed site-directed mutagenesis of the cysteine residues (C)-235, C275, and C578 of the *gcd* gene, which made the interaction with *dsb*A1 less pronounced and affected the amount of DAPG produced to some extent.

Li et al. [186], also using *P. fluorescens* 2P24 as an example, demonstrated the influence of two other factors, the outer membrane protein gene *opr*F and the sigma factor *sig*X, on DAPG production. SigX was located in the genome of the strain directly upstream of the *opr*F gene and was a positive regulator of *opr*F transcription. The Δ*opr*F mutant produced approximately 1.5-fold more DAPG than the control, and Δ*sig*X produced approximately 30% of the amount of DAPG produced by the parental strain.

Zhang et al. [184] and Yan et al. [173], using the strain *P. fluorescens* strain 2P24, demonstrated that modified (mutant) variants that produce large amounts of DAPG grow more slowly due to the increased metabolic costs in the cells.

### 5.4. DAPG in Biotechnology

DAPG is a broad-spectrum antibiotic that is effective against yeast (*Saccharomyces* [195,196], *Candida* [197]), fungi (*Fusarium* [198], *Rhizoctonia solani*, *Sclerotium rolfsii*, *Macrophomina phaseolina* [199], *Pythium* [131], *Gaeumannomyces graminis* [195,200], *Thielaviopsis* [201]), bacteria (*Staphylococcus aureus* [202,203], *Ralstonia* [199], *Xanthomonas* [204]), and nematodes (*Meloidogyne incognita* [205,206] and *M. javanica* [207], *Globodera rostochiensis* [208]).

Natural producers of DAPG include *Pseudomonas protegens* CL145A (ATCC 55799) [209,210], Pf-5 [211], *P. fluorescens* NRRL B-21806 and B-21807 [212], and *P. congelans* DSM 21663 [148].

The *Phl* gene cluster and a set of constructs using these sequences have been patented [213]. The sequence donor was *P. fluorescens* Q2-87, and the purpose of these constructs was twofold: (1) *Pseudomonas* strains lacking the ability to produce DAPG to acquire this ability, and (2) *Pseudomonas* strains already capable of producing DAPG to enhance their properties as producers. The modified strains were supposed to be used for plant defense against fungal pathogens.

The use of genetically modified DAPG producers in biotechnology has garnered significant attention, with various studies conducted to evaluate their impact on the biodiversity of natural microbial communities. For instance, research has shown that DAPG overproducer based on *P. fluorescens* F113 could positively affect mycorrhizal symbiosis and stimulate the development of micelles of the symbiotic fungus *Glomus mosseae* on maize roots [214]. On the other hand, it has also been demonstrated that this overproducer has no discernible effect on the indigenous microbial population in the rhizosphere of sugar beet [215].

“Phl” and “Plt” overproducers derived from *P. fluorescens* CHA0 impacted the composition of the microbial communities in cucumber, albeit temporary [216,217]. Niemann et al. [218] demonstrated that the addition of *P. fluorescens* CHA0 and its modified variant decreased the population of the nitrogen-fixing symbiotic *Sinorhizobium meliloti* within the soil microbiome.

Modifying *Pseudomonas* strains to enhance their properties as biocontrol strains is a well-known practice. Huang et al. [219] genetically modified a natural DAPG-producing strain of *Pseudomonas fluorescens* Q8r1-96 by integrating a plasmid containing the *phz* operon, which is responsible for phenazine production. This engineered strain is anticipated to be effective against *Rhizoctonia*, *Gaeumannomyces graminis* or *Pythium*.

Zhang et al. [29] incorporated a genomic island containing NiF (nitrogen fixation) genes into *P. protegens* Pf-5. The donor of the genomic island was *Pseudomonas stutzeri* strain DSM4166. Through markerless mutagenesis of the *ret*S gene, the resulting Pf-5-NiF strain exhibited an increased level of DAPG production [156].

## 6. Conclusions

This article focuses on antibiotics produced by *Pseudomonas* strains, namely mupirocin, gluconic acid, PRN, and DAPG. Each section on an antibiotic covers aspects like the genetic structure of the biosynthesis pathway, the regulation of this process, and the application of the antibiotic in medical or agricultural biotechnology.

Even with the great progress in understanding the genetics of antibiotic-producing pseudomonads, a number of issues remain challenging for the future generations. For instance, it would be interesting to develop a method for the production of mupirocin consisting of only pseudomonic acid C. Due to its chemical structure, this pseudomonic acid is more stable than the others (A, B, D) and has the same antibiotic properties as PA-A. To date, attempts to obtain high yields of PA-C alone have not been successful.

Although the interest in antibiotics of *Pseudomonas* bacteria has persisted over the past century, many uncertainties still surround the regulation of the biosynthetic pathways of these compounds. Furthermore, the challenge of obtaining stable producers that yield higher quantities of these antibiotics continues to be a pertinent concern in modern biotechnology.

In the field of basic science, there are documented instances where producers have been successfully modified to provide high yields of the target product. However, only a few of them have been patented for biotechnological purposes. It is of interest to use modified producers in practical settings, not only in the laboratory, because in many cases genetic constructs show instability when experiments are scaled up.

Future research on antibiotics of pseudomonads can follow several main directions. First, there is biotechnological interest in improving the efficiency of antibiotic production using already-known producers and searching for new ones [123,199]. Second, there are proposals to combine antibiotic-producing strains with other useful strains, creating consortia [162]. Such work would involve studying the compatibility of strains and working out the conditions that enhance their joint productivity. Third, in both medicine and plant protection technologies, deciphering the molecular basis of pathogen sensitivity to pseudomonad antibiotics is necessary [162,220]. Since the problem of pathogen resistance to antibiotics remains relevant, special attention should be paid to the development of drug-delivery strategies, combining pseudomonad antibiotics with other agents such as anesthetics and other antibiotics [220].

This review provides a comprehensive summary of the genetic organization of the antibiotic biosynthesis pathways in *Pseudomonas* strains, appealing to both molecular biologists and biotechnologists.

## Figures and Tables

**Table 1:** The known mixtures of pseudomonic acids produced by industrial strains of *P. fluorescens.*.

Producer Strain	Mixture Composition	Component Ratio	Reference
*Pseudomonas fluorescens* NCIB 10586	Pseudomonic acid A, its isomer with a cis double bond in the cis position between the carbon atoms C2 and C3, and pseudomonic acid B	4.5:4.5:1	[33,34]
	Pseudomonic acids A, B, C and D	90:8:1:1	[35]
*Pseudomonas fluorescens* Y-11633	Pseudomonic acids A and B, and two components with unknown structures	9:0.5:0.5	[36]
*Pseudomonas* sp. No 19/26	The main component is pseudomonic acid A, and minor amounts of pseudomonic acids B and C are also present	ND	[36]

**Table 2:** Structure of the mupirocin biosynthesis gene cluster.

Module	Composition of Module	Corresponding Genes	Reference
I	Large polyketide synthases (PKS)	Type I multifunctional gene *mmp*A	[44,45]
		Type I multifunctional gene *mmp*D	
	Small PKS	Trans-acyltransferase *mmp*C	
	ORF	*mup*A	
	ORF	*mup*B	
II	Small PKSs	*mmp*E	[40,41]
		*mmp*F	
	27 single ORFs	*mup*C-X and *macp*A-E	

**Table 3:** Role of *mup*I and *mup*R in the regulation of mupirocin production.

Gene	Role in Mupirocin Production	Reference
*mup*I	Essential for generating of N-(3-oxodecanoyl) homoserine lactone (3-O-C10-HSL)	[53]
*mup*R	3-O-C10-HSL binds to *Mup*R, thereby activating the promoter	

**Table 4:** Commercial preparations based on PRN derivatives.

Brand Name	The Main Active Ingredient	Additional Ingredients	Target Organisms
SAPHIRE	Fludioxonil, ND	-	*Microdochium* spp., *Fusarium*, *Septoria*
BERET	Fludioxonil, 25 g/L	-	*Microdochium* spp., *Fusarium*, *Septoria*
CELEST	Fludioxonil, 25 g/L	Thiamethoxam, 262.5 g/L, difenoconazole, 25 g/L	*Microdochium nivale, Fusarium*, *Septoria*
MAXIM	Fludioxonil, 25 g/L	Mefenoxam, 10 g/L	*Rhizoctonia solani*, *Fusarium, Helminthosporium*
GALBAS	Fenpiclonil, ND	-	*Rhizoctonia solani*, *Helminthosporium solani*, *Fusarium solani* f. sp. *coeruleum*, *Fusarium sulphureum*

## Data Availability

Not applicable.
